# Deficiency of iNOS-derived NO accelerates lipid accumulation-independent liver fibrosis in non-alcoholic steatohepatitis mouse model

**DOI:** 10.1186/s12876-015-0269-3

**Published:** 2015-04-01

**Authors:** Yuichi Nozaki, Koji Fujita, Koichiro Wada, Masato Yoneda, Takaomi Kessoku, Yoshiyasu Shinohara, Kento Imajo, Yuji Ogawa, Makoto Nakamuta, Satoru Saito, Naohiko Masaki, Yoji Nagashima, Yasuo Terauchi, Atsushi Nakajima

**Affiliations:** 1 Division of Gastroenterology, Yokohama City University Graduate School of Medicine, 3-9 Fuku-ura, Kanazawa-ku, Yokohama, 236-0004 Japan; 2 Department of Gastroenterology, National Center for Global Health and Medicine, 1-21-1, Toyama, Shinjuku-ku, Tokyo, 162-8655 Japan; 3 Department of Pharmacology, Graduate School of Dentistry, Osaka University, 1-8 Yamadaoka, Suita, Osaka, 565-0871 Japan; 4 Department of Gastroenterology, Kyushu Medical Center, National Hospital Organization, 1-8-1, Jigyohama, Chuo-ku, Fukuoka, 810-8563 Japan; 5 The Research Center for Hepatitis and Immunology, National Center for Global Health and Medicine, 1-7-1, Konodai, Ichikawa, 272-8516 Japan; 6 Department of Molecular Pathology, Yokohama City University Graduate School of Medicine, 3-9 Fuku-ura, Kanazawa-ku, Yokohama, 236-0004 Japan; 7 Department of Endocrinology and Metabolism, Yokohama City University Graduate School of Medicine, 3-9 Fuku-ura, Kanazawa-ku, Yokohama, 236-0004 Japan

**Keywords:** NOS, NASH, Inflammation, Fibrosis, NF-kB

## Abstract

**Background:**

Although many of the factors and molecules closely associated with non-alcoholic steatohepatitis (NASH) have been reported, the role of inducible nitric oxide synthase (iNOS)-derived nitric oxide (NO) on the progression of NASH remains unclear. We therefore investigated the role of iNOS-derived NO in NASH pathogenesis with a long-term follow-up study using systemic iNOS-knockout mice under high-fat diet (HFD) conditions.

**Methods:**

iNOS-knockout and wild-type mice were fed a basal or HFD for 10 or 48 weeks. Lipid accumulation, fibrosis, and inflammation were evaluated, and various factors and molecules closely associated with NASH were analyzed.

**Results:**

Marked fibrosis and inflammation (indicators of NASH) were observed in the livers of iNOS-knockout mice compared to wild-type mice after 48 weeks of a HFD; however, lipid accumulation in iNOS-knockout mice livers was less than in the wild-type. Increased expressions of various cytokines that are transcriptionally controlled by NF-kB in iNOS-deficient mice livers were observed during HFD conditions.

**Conclusions:**

iNOS-derived NO may play a protective role against the progression to NASH during an HFD by preventing fibrosis and inflammation, which are mediated by NF-kB activation in Kupffer cells. A lack of iNOS-derived NO accelerates progression to NASH without excessive lipid accumulation.

## Background

Non-alcoholic fatty liver disease (NAFLD) is the most common liver disease, associated with systemic insulin resistance (IR) and chronic liver inflammation, which includes non-alcoholic fatty liver (NAFL) and non-alcoholic steatohepatitis (NASH). NAFL represents the first phase of NASH, which is characterized by steatosis, and can then develop into fatty liver disease with associated inflammation [1]. Although NASH is thought to lead to liver fibrosis, cirrhosis and hepatocellular carcinoma, resulting in increased morbidity and mortality, the pathogenesis of NASH—such as the progression of hepatic NAFL to NASH—remains unclear.

We have previously reported that several potential mechanisms are involved in the pathogenesis of NASH [2-4]. Namely, we clearly showed that decrease in very low-density lipoprotein (VLDL) synthesis caused lipid accumulation in liver [2,3] and that leptin-mediated CD14 up-regulation aggravated inflammation of liver [4]. However, other factors associated with NASH progression remain to be determined.

Nitric oxide (NO) plays an important role in liver disease associated with IR and inflammation, and it has been reported that chronic and systemic deficiency of inducible nitric oxide synthase (iNOS) ameliorated high fat diet (HFD)-induced whole-body IR [5-7]. Therefore, iNOS is considered to be a target for the prevention of fat accumulation in the liver, such as is associated with NAFL. However, in liver inflammation and fibrosis, NO is thought to have a protective effect against various types of damage which may occur. Generated NO maintains the hepatic microcirculation [8,9], and inhibition of NO generation leads to increased hepatic damage [10,11]. In the pathogenesis of NAFLD/NASH, the role of NO is debatable: while the NO-AMP-activated protein kinase (AMPK)-peroxisome proliferator-activated receptor α (PPARα) signaling pathway is crucial for the control of hepatic fatty acid oxidation [12], the excess production of NO content, which leads to nitrosative stress, is correlated with the severity of NAFLD [13]. Because of the conflicting effects of NO on the liver, the exact roles of iNOS and generated NO in the pathogenesis of NASH has not yet been elucidated.

In this study, we investigated the role of iNOS-derived NO in the pathogenesis of NASH in a long-term follow-up study using systemic iNOS-knockout mice under HFD conditions.

## Methods

### Animal treatment and procedures

We purchased a C57BL/6 J backcrossed C57BL/6 J-Nos2tm1Lau colony from Jackson Labs (Bar Harbor, ME 04609, USA) to create male congenic wild-type (*iNOS+/+*) and *iNOS* knockout (*iNOS−/−*) mice. Mice were allowed free access to food and tap water throughout the acclimatization and experimental periods.

From 6 weeks of age, each strain of mice were fed a basal diet (BD) or a HFD. Forty-eight mice were dividing into 4 groups: [1] *iNOS+/+*/BD; [2] *iNOS−/−*/BD; [3] *iNOS+/+*/HFD; and [4] *iNOS−/−*/HFD. Two experimental periods of 10 and 48 weeks were examined using 6 mice from each group for each period. A HFD was administered using high-fat diet 32 containing 20% protein, 60% fat, and 20% carbohydrate in powdered form from Japan CLEA (Tokyo, Japan) and a BD containing 22% protein, 6% fat, and 47% carbohydrate was administered using MF from Oriental Yeast Co., Ltd. (Tokyo, Japan). The precise contents of these feeds were as previously described [2]. All animals were treated humanely according to the guidelines from the National Institutes of Health and the AERI-BBRI Animal Care and Use Committee. Animal protocols were approved by the Yokohama City University Medical School guidelines for the care and use of laboratory animals.

### Measurement of plasma and serum biochemical markers

Serum alanine aminotransferase (ALT) was measured using Spotchem SP-4410 (Arklay Co., Kyoto, Japan). Total serum cholesterol, triacylglycerol (TG) and plasma lipoprotein levels were analyzed by an online dual-enzymatic method for simultaneous quantification of cholesterol and TG by high-performance liquid chromatography according to a procedure previously described [2,14,15]. Fasting plasma glucose, serum levels of fasting insulin, leptin, adiponectin and non-esterified fatty acid (NEFA) were determined using a Glutest Pro kit (Sanwa Kagaku Kenkyusyo Co., Nagoya, Japan), an Ultra Sensitive Insulin ELISA kit (Biochemical Research Laboratory, Morinaga Milk Industry Co., Tokyo, Japan), an ELISA Mouse Leptin kit (Biochemical Research Laboratory, Morinaga Milk Industry Co., Tokyo, Japan), a Mouse/Rat Adiponectin ELISA kit (Otsuka Pharmaceutical Co., Tokyo, Japan), NEFA C-Test kit (Wako Pure Chemical Industries Co., Osaka, Japan), respectively. Blood IR was estimated using the homeostasis model assessment of IR (HOMA-IR) derived from the following equation: IR = fasting plasma glucose level mg/dl × fasting serum insulin level ng/ml /22.5.

### Insulin tolerance test (ITT)

Mice fed freely before being fasted for the study. They were intraperitoneally challenged with 0.75 mU/g body weight human insulin (Novolin R; Novo Nordisk, Denmark). Blood samples were collected to measure glucose levels at 0, 20, 40, 60, 80, 100 and 120 minutes after the insulin injection [16].

### Liver histopathological and immunohistochemical evaluations

Liver samples were excised and embedded in Tissue-Tek OCT compound (Sakura Finetek USA Inc, Torrance, CA, USA) and paraffin for histological analysis. Formalin-fixed and paraffin-embedded sections were processed routinely with hematoxylin and eosin (H&E) and Masson’s trichrome staining. For evaluation of fat deposition, the OCT-embedded samples were stained with oil-red O. For quantification of liver collagen content, 50 fields were microscopically examined at a 40× magnification using a grid of 0.0625 mm2 with 100 points in Masson’s trichrome-stained preparations. The samples were also stained with monoclonal anti-actin, α-smooth muscle antibodies (α-SMA) (Sigma-Aldrich, St Louis, MO, USA) and phospho-nuclear factor kappa B (NF-kB) antibodies (Cell Signaling Technology Japan, K.K., Tokyo, Japan). Immunofluorescence was performed using 7-μm cryostat liver sections. The sections were incubated with primary antibodies and stained with Alexa Fluoro-conjugated secondary antibodies (Cell Signaling Technology, Inc, Japan). The primary antibodies used were phospho-nuclear factor kappa B (NF-kB) antibodies (Cell Signaling Technology Japan, K.K., Tokyo, Japan), F4/80 (Santa Cruz Biotechnology, Inc, Santa Cruz, CA, USA), and desmin antibodies (Wako Pure Chemical Industries Co., Osaka, Japan) [4].

### Liver histology and scoring systems

All histopathological findings were scored by the same pathologists (Y.N. and S.M.), who were unaware of the treatment that the animals had received. The histological features were grouped into 3 broad categories: steatosis; inflammation and hepatocyte ballooning; and fibrosis, for use with the NAFLD activity score (NAS) [17]. The system of evaluation, which has previously been described [18], is detailed in Table *1*.

**Table 1:** **Primer sequences used for real-time PCR analysis**

Gene	Primer sense	Primer antisense
SREBP-1c	CAGCTATTGGCCTTCCTCAG	CCTGGACCATTTTAGCCTCA
ChREBP	CCTCACTTCACTGTGCCTCA	ACAGGGGTTGTTGTCTCTGG
DGAT2	AGGCCCTATTTGGCTACGTT	GATGCCTCCAGACATCAGGT
PPAR-α	GTCCTCAGTGCTTCCAGAGG	GGTCACCTACGAGTGGCATT
MTP	GCCCTAGTCAGGAAGCTGTG	CCAGCAGGTACATTGTGGTG
IL-1	CCCGTCCTTAAAGCTGTCTG	AATTGGAATCCAGGGGAAAC
TNF-α	TATGGCTCAGGGTCCAACTC	CTCCCTTTGCAGAACTCAGG
TGF-β	TGAGTGGCTGTCTTTTGACG	AGCCCTGTATTCCGTCTCCT
PDGF	CGTTCACTTCCGGTTCATTT	GTGGAGGAGCAGACTGAAGG
TIMP-1	CTGTTGGCTGTGAGGAATGCA	TTCAGAGCCTTGGAGGAGCTG
TIMP-2	CACAGACTTCAGCGAATGGA	CTTGGGAAGCTTGAGAGTGG
CollagenI	GAGCGGAGAGTACTGGATCG	GTTCGGGCTGATGTACCAGT

### Measurement of liver triglyceride and fatty acid content

Liver samples were homogenized in 50 mM Tris/HCl buffer, pH 7.4, containing 150 mM NaCl, 1 mM EDTA and 1 mM PMSF. TG levels were analyzed enzymatically using a diagnostic kit (Infinity™, Thermo DMA, Arlington, TX, USA). Liver NEFA levels were determined by an acyl CoA synthetase and acyl CoA oxidase enzyme method with a commercial kit (NEFA C-Test; Wako Pure Chemical Industries, Co., Osaka, Japan), after a chloroform/methanol extraction according to the method described by Folch et al. [19].

### Liver NO assay

NO concentrations in the liver were determined using a nitrate/nitrite colorimetric assay kit (Cayman Chemical, Ann Arbor, Michigan 48108, USA). This assay is based on the enzymatic conversion of nitrate to nitrite by nitrate reductase. We constructed a standard curve using NO standards [20].

### Liver microsomal triglyceride transfer protein (MTP) activity assay

MTP activity was measured using an MTP assay kit (Roar Biochemical, New York, NY, USA) according to a previously described method [21,22].

### Quantification of gene expressions by real-time RT-PCR

Total RNA was isolated from the samples using an RNeasy Mini kit (Qiagen GmbH., Hilden, Germany, Cat NO. 74126), and reverse transcription to produce cDNA using a TaqMan Gold RT-PCR Kit (Applied Biosystems, Foster City, CA, USA), were performed according to the manufacturer’s instructions as previously described [2].

Hepatic mRNA levels of several markers, as well as of the housekeeping gene, β-actin, in the liver tissue were determined using fluorescence-based real-time RT-PCR on an ABI PRISM 7700 Sequence Detection System (Applied Biosystems, Foster City, CA, USA). Real-time RT-PCR was performed using the TaqMan and Power SYBR® Green PCR Master Mix reagent, according to the manufacturer’s instructions (Applied Biosystems, Foster City, CA, USA). Values were normalized to the expression level of the endogenous control, β-actin. The gene expression ratio was determined using data from WB mice as the control group. The Probe and primer pair specific for β-actin and MTP was purchased from Applied Biosystems. Primer sequences are listed in Table 1.

### Electrophoretic mobility shift assay

Electrophoretic mobility shift assays (EMSA) were performed according to a previously described method [23]. Briefly, nuclear extracts from liver tissue were prepared, and then gel shift assays using a NF-kB consensus oligonucleotide (Promega, Madison, WI, USA) were performed. Samples were separated using 4% polyacrylamide gel electrophoresis, before the gels were dried and exposed to radiograph film.

### Statistical analysis

Statistical analyses were performed using SPSS for Windows, version 12. All results are expressed as the mean ± SEM. Statistical comparisons were made using the Student *t*-test or Scheffe’s method after an analysis of variance. Results of *p* < 0.05 were considered statistically significant.

## Results

### iNOS-derived NO promotes liver steatosis in HFD mice

**Figure 1:**
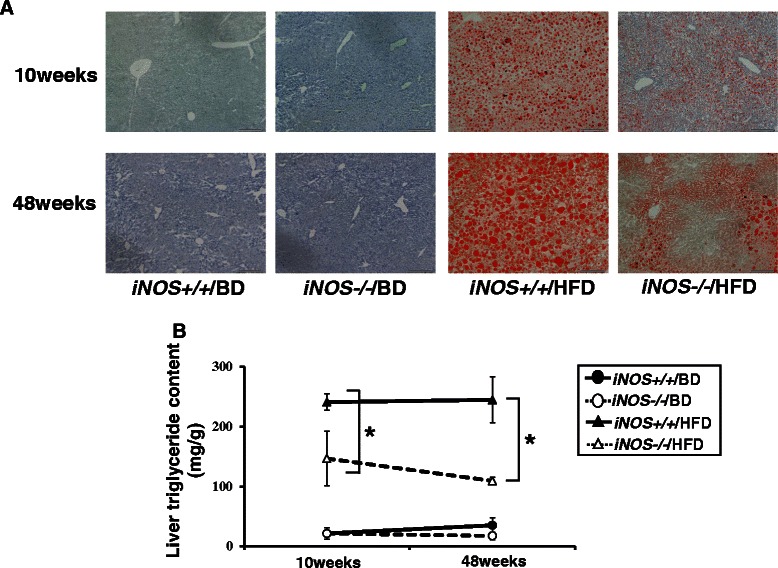
**Analysis of liver steatosis. (A)** Oil-red O staining (red color) shows lipid deposits in liver samples. At both 10 and 48 weeks, macrovesicular steatosis was visible in *iNOS+/+*/HFD mice and microvesicular steatosis was visible in *iNOS−/−*/HFD mice. Scale bar, 200 μm. **(B)** Liver TG content was significantly higher in *iNOS+/+*/HFD mice than in *iNOS−/−*/HFD mice at both 10 and 48 weeks. (Data are expressed as the mean ± SEM. *p < 0.05, represents significant difference between *iNOS+/+*/HFD mice and *iNOS−/−*/HFD mice).

In the NAFLD/HFD model, steatosis was observed after 10 weeks of HFD feed. Typical fibrosis was observed after 36–48 weeks of HFD feed [2,4]. While *iNOS−/−*/HFD mice showed microvesicular steatosis, *iNOS+/+*/HFD mice showed macrovesicular steatosis in oil-red O staining liver samples at both 10 and 48 weeks (Figure 1A). Liver TG content was significantly higher in *iNOS+/+*/HFD mice compared with *iNOS−/−*/HFD mice at both 10 and 48 weeks (Figure 1B). The steatosis grade using NAS scores showed a tendency for *iNOS+/+*/HFD mice to be higher than *iNOS−/−*/HFD mice at both periods, but not significantly (Table 2). The results suggested that iNOS-derived NO promoted liver steatosis in HFD mice during the experimental period.

**Table 2:** **Histological scores of livers by using NAFLD activity score (NAS)** ^**17)**^

			10 W model				48 W model			
Item	Definition	Score	*iNOS+/+*	*iNOS−/−*	*iNOS+/+*	*iNOS−/−*	*iNOS+/+*	*iNOS−/−*	*iNOS+/+*	*iNOS−/−*
			BD	BD	HFD	HFD	BD	BD	HFD	HFD
Steatosis										
Grade	Parenchymal involvement									
	<5%	0	6	6	0	0	6	6	0	0
	5-33%	1	0	0	4	6	0	0	3	6
	33-66%	2	0	0	2	0	0	0	3	0
	>66%	3	0	0	0	0	0	0	0	0
		Average	0.00	0.00	1.33	1.00	0.00	0.00	1.50	1.00
Inflammation										
Lobular inflammation	Assessment of all inflammatory foci									
	No foci	0	6	6	5	4	6	6	0	0
	<2 foci per 200 × field	1	0	0	1	3	0	0	5	2
	2-4 foci per 200 × field	2	0	0	0	0	0	0	1	4
	>4foci per 200 × field	3	0	0	0	0	0	0	0	0
Liver cell injury	Ballooning									
	None	0	6	6	0	0	6	6	0	0
	Few balloon cells	1	0	0	6	6	0	0	4	0
	Many cells/prominent balloooning	2	0	0	0	0	0	0	2	6
		Average	0.00	0.00	1.17	1.33	0.00	0.00	2.50^*^	3.67^*^
Fibrosis										
Stage	Method of Brunt									
	None	0	6	6	6	6	6	6	0	0
	Perivenular/perisinusoidal fibrosis	1	0	0	0	0	0	0	6	0
	Combine pericellular portal fibrosis	2	0	0	0	0	0	0	0	5
	Septal/bridging fibrosis	3	0	0	0	0	0	0	0	1
	Cirrhosis	4	0	0	0	0	0	0	0	0
		Average	0.00	0.00	0.00	0.00	0.00	0.00	1.00^**^	2.17^**^

BD, basal diet; HFD, high fat diet;

Significant differences exist between *iNOS+/+*/HFD vs *iNOS−/−*/HFD for listed parameters at ^*^p < .05 level and ^**^p < .01 level.

### iNOS-derived NO protects liver inflammation and liver fibrosis in 48-week HFD mice

**Figure 2:**
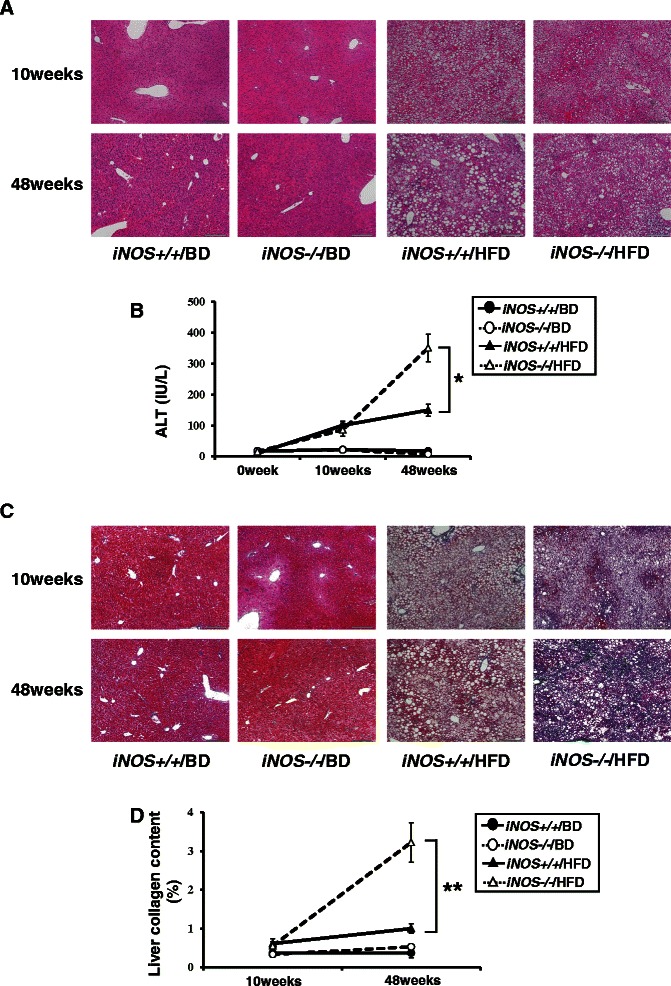
**Analysis of liver inflammation and liver fibrosis. (A)** Liver samples were stained with H&E. By evaluating the amount of inflammatory foci per field and ballooning, *iNOS−/−*/HFD mice showed more severe liver inflammation than *iNOS+/+*/HFD mice at 48 weeks. Scale bar, 200 μm. **(B)** Serum ALT levels were significantly higher in *iNOS−/−*/HFD mice than in *iNOS+/+*/HFD mice at 48 weeks. **(C)** Liver samples were stained with Masson’s trichrome. No obvious fibrosis is visible in the liver specimens from any group at 10 weeks. While only perivenular and perisinusoidal fibrosis was visible in *iNOS+/+*/HFD mice, combined pericellular portal fibrosis and bridging fibrosis was visible in *iNOS−/−*/HFD mice at 48 weeks. Scale bar, 200 μm. **(D)** Liver collagen content was significantly higher in *iNOS−/−*/HFD mice than in *iNOS+/+*/HFD mice at 48 weeks. (Data are expressed as the mean ± SEM. * *p* < 0.05 and ** *p* < 0.01, represents significant difference between *iNOS+/+*/HFD mice and *iNOS−/−*/HFD mice).

In the 48-week HFD mice groups, analysis of liver inflammation using H&E staining revealed histological inflammation scores which were significantly higher in *iNOS−/−*/HFD mice compared with *iNOS+/+*/HFD (Table 2, Figure 2A). Serum ALT values were also significantly higher in *iNOS−/−*/HFD mice compared with *iNOS+/+*/HFD mice (Figure 2B).

Analysis of liver fibrosis from liver samples subject to Masson’s trichrome staining using the Brunt classification revealed that the histological scores and the liver collagen content (which represent liver fibrosis) were significantly higher in *iNOS−/−*/HFD mice compared with *iNOS+/+*/HFD mice after 48 weeks of HFD feed (Table 2, Figure 2C and D). In contrast, both the *iNOS+/+*/HFD and the *iNOS−/−*/HFD mice showed little or no liver inflammation or fibrosis after 10 weeks of HFD feed. (Table 2, Figure 2A and C).

### Comparison of parameters associated with pathogenesis of NAFLD between wild-type and iNOS knockout mice in HFD conditions

**Figure 3:**
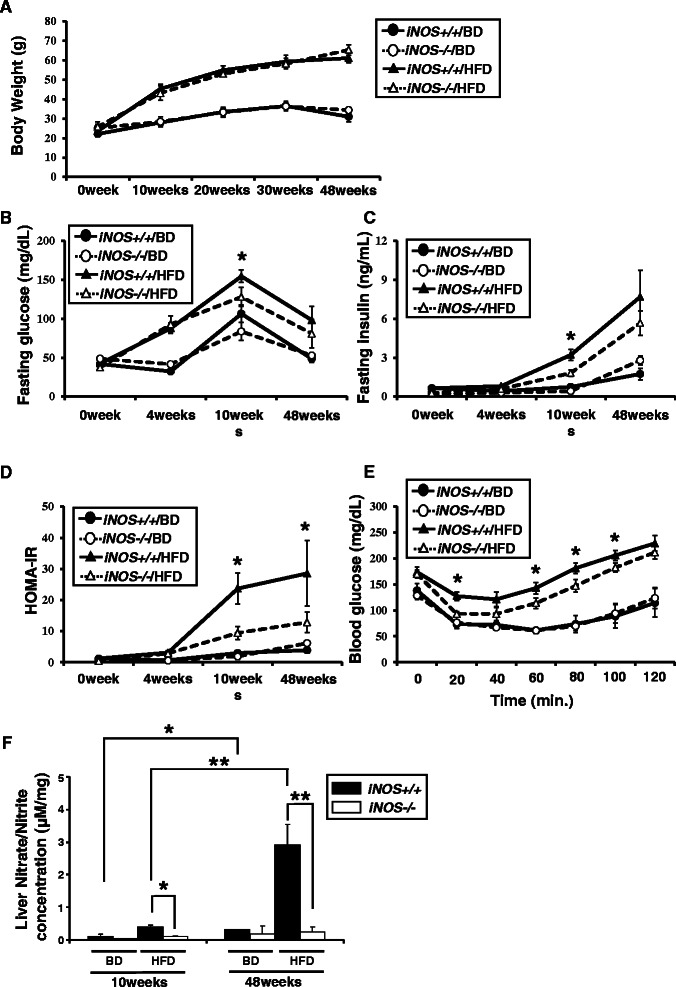
**Analysis of factors associated with the pathogenesis of NAFLD: body weight, systemic insulin resistance and liver NO metabolites. (A)** No significant difference was observed between *iNOS+/+*/HFD and *iNOS−/−*/HFD mice throughout the experimental period. **(B, C and D)** Fasting glucose and fasting insulin levels were significantly higher in *iNOS+/+*/HFD mice than in *iNOS−/−*/HFD mice at 10 weeks. HOMA-IR levels were significantly higher in *iNOS+/+*/HFD mice than in *iNOS−/−*/HFD mice at both 10 and 48 weeks. **(E)** Decreases in blood glucose were significantly greater in *iNOS+/+*/HFD mice than in *iNOS−/−*/HFD mice at 20, 60, 80 and 100 minutes after insulin injection. **(F)** The liver nitrate/nitrite concentrations in *iNOS+/+*/HFD mice were higher at 48 weeks than at 10 weeks; and other groups showed little concentrations at both 10 and 48 weeks. Liver nitrate/nitrite concentrations were significantly higher in *iNOS+/+*/HFD mice than in *iNOS−/−*/HFD mice at both 10 and 48 weeks. (Data are expressed as the mean ± SEM. * *p* < 0.05 and ** *p* < 0.01, represents significant difference between *iNOS+/+*/HFD mice and *iNOS−/−*/HFD mice).

Body weights of HFD mice increased during the experimental period, but no significant differences between *iNOS+/+*/HFD and *iNOS−/−*/HFD mice were observed (Figure 3A). Analysis of several parameters associated with the pathogenesis of NAFLD revealed that the liver weights were higher in *iNOS+/+*/HFD mice compared with *iNOS−/−*/HFD mice at 10 weeks; and that serum adiponectin, leptin and NEFA levels were significantly higher in *iNOS−/−*/HFD mice compared with *iNOS+/+*/HFD mice at 48 weeks. Liver NEFA content was significantly higher in *iNOS−/−*/HFD mice than in *iNOS+/+*/HFD mice at 48 weeks. No significant difference showed at 10 weeks (Table 3).

**Table 3:** **Characteristics of mice in 10 and 48 week model**

	10 W model				48 W model			
	*iNOS+/+*	*iNOS−/−*	*iNOS+/+*	*iNOS−/−*	*iNOS+/+*	*iNOS−/−*	*iNOS+/+*	*iNOS−/−*
	BD	BD	HFD	HFD	BD	BD	HFD	HFD
Number of animals	6	6	6	6	6	6	6	6
Liver weight (g)	1.25	1.12	3.11^*^	2.19^*^	1.22	1.45	5.92	5.38
	±0.07	±0.05	±0.18	±0.32	±0.24	±0.11	±0.61	±0.38
Visceral fat weight (g/100 g BW)	2.24	1.98	5.10	5.26	2.60	2.77	3.52	3.49
	±0.15	±0.17	±0.32	±0.50	±1.18	±0.40	±0.04	±0.52
Subcutaneous fat weight (g/100 g BW)	0.64	0.74	4.22	3.81	1.17	0.91	4.65	4.91
	±0.11	±0.12	±0.20	±0.37	±0.53	±0.20	±0.23	±0.66
Mesenterial fat weight (g/100 g BW)	0.36	0.57	3.02	3.09	1.10	0.56	1.39	1.54
	±0.03	±0.08	±0.19	±0.14	±0.91	±0.04	±0.07	±0.02
Serum cholesterol (mg/dL)	86.4	82.6	167.7	133.3	88.3	67.7	230.6	283.7
	±3.6	±5.7	±6.5	±16.6	±9.7	±4.6	±24.0	±10.4
Serum TG (mg/dL)	30.1	23.8	40.1	35.0	26.8	26.4	16.5	17.5
	±3.1	±5.0	±4.5	±1.9	±3.1	±1.5	±0.4	±2.6
Serum VLDL-TG (pg/mL)	12.7	11.7	16.4	14.7	11.6	14.6	3.5	4.0
	±4.2	±1.8	±2.4	±1.2	±1.3	±1.6	±0.1	±0.6
Serum adiponectin (μg/mL)	15.0	15.1	13.3	13.2	11.0	10.7	6.9^*^	8.5^*^
	±1.4	±1.3	±0.6	±0.7	±0.7	±0.3	±0.4	±0.1
Serum leptin (ng/mL)	2.6	1.4	29.8	27.7	3.1	3.2	60.9^*^	130.7^*^
	±0.5	±0.4	±1.0	±1.1	±0.6	±0.5	±12.1	±27.4
Serum NEFA (mEq/L)	1.0	0.9	1.5	1.6	0.9	1.3	1.7^*^	2.6^*^
	±0.1	±0.1	±0.1	±0.1	±0.2	±0.2	±0.1	±0.2
Liver NEFA contents (μEq/g)	13.9	18.3	12.4	12.6	10.3	8.4	10.7	17.6^*^
	±2.5	±6.0	±1.4	±1.4	±1.1	±0.8	±1.6	±2.7

BD, basal diet; HFD, high fat diet; TG, triglyceride; VLDL-TG, very low density lipoprotein-TG;

NEFA, nonesterified fatty acid

Data are expressed as mean ± SEM.

Significant differences exist between *iNOS+/+*/HFD vs *iNOS−/c*/HFD for listed parameters at ^*^p < .05 level.

Analysis of systemic IR revealed that the fasting glucose and fasting insulin levels were significantly higher in *iNOS+/+*/HFD mice compared with *iNOS−/−*/HFD mice at 10 weeks and HOMA-IR levels were significantly higher in *iNOS+/+*/HFD mice compared with *iNOS−/−*/HFD mice at both 10 and 48 weeks (Figure 3B, C and D).

The ITT results at 10 weeks showed that the systemic response to insulin injection was significantly lower in *iNOS+/+*/HFD mice compared with *iNOS−/−*/HFD mice (Figure 3E).

Liver nitrate/nitrite concentrations were significantly higher in *iNOS+/+*/HFD mice compared with *iNOS−/−*/HFD mice. Interestingly, the increased liver NO generation was time-dependent and HFD-induced phenomena. In addition, the increased NO generation during HFD conditions is considered to be derived from iNOS, because the iNOS knockout mice did not show an increase in NO metabolites during HFD conditions. The liver nitrate/nitrite concentrations in *iNOS+/+*/HFD mice were higher at 48 weeks than at 10 weeks (Figure 3F).

### Analysis of the mechanisms of liver steatosis, inflammation and fibrosis

**Figure 4:**
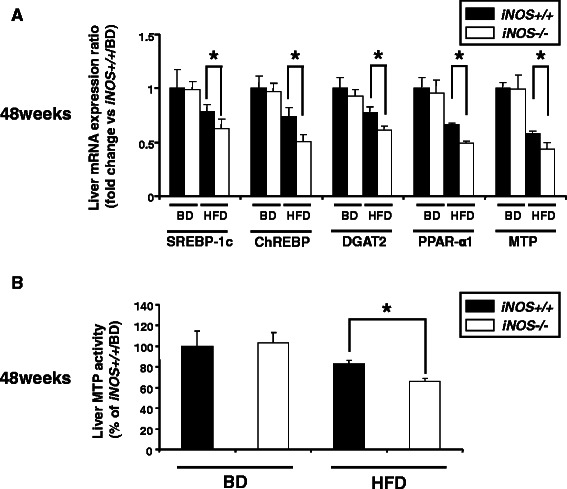
**Analysis of the mechanisms of liver steatosis. (A)** Liver SREBP-1c, ChREBP, DGAT2, PPAR-α1 and MTP mRNA expression levels were all significantly lower in *iNOS−/−*/HFD mice than in *iNOS+/+*/HFD mice at 48 weeks. **(B)** Liver MTP activity was significantly lower in *iNOS−/−*/HFD mice than in *iNOS+/+*/HFD mice at 48 weeks. (Data are expressed as the mean ± SEM. * *p* < 0.05, represents significant difference between *iNOS+/+*/HFD mice and *iNOS−/−*/HFD mice).

At 48 weeks, it was observed that all sterol regulatory element binding protein-1c (SREBP-1c), carbohydrate response element binding protein (ChREBP), diacylglycerol acyltransferase2 (DGAT2), PPAR-α1 and MTP were significantly down-regulated by the HFD fed mice. Further decreases in mRNA of those molecules were also observed in *iNOS−/−*/HFD mice compared to those of the *iNOS+/+*/HFD mice (Figure 4A). Analysis of liver MTP activity was consistent with the results of liver mRNA expression levels (Figure 4B).

**Figure 5:**
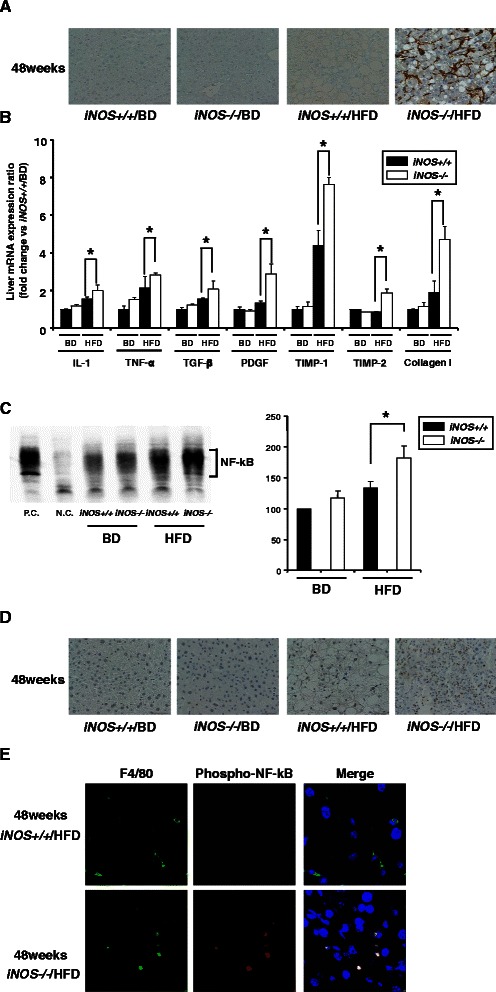
**Analysis of the mechanisms of liver inflammation and liver fibrosis, and of the effects of NF-kB activity inhibitor on iNOS knockout/HFD mice livers at 48 weeks. (A)** Liver samples were stained with α-SMA antibodies. Pericellular staining was visible only in *iNOS−/−*/HFD mice at 48 weeks. Scale bar, 50 μm. **(B)** Liver IL-1, TGF-β, PDGF, TIMP-1, TIMP-2 and collagen I mRNA expression levels were significantly higher in *iNOS−/−*/HFD mice than in *iNOS+/+*/HFD mice at 48 weeks. **(C)** Analysis of liver NF-kB activity by EMSA showed that the NF-kB band of *iNOS−/−*/HFD mice at 48 weeks was enhanced compared with *iNOS+/+*/HFD mice. P.C.: positive control using HeLa cells. N.C.: negative control. **(D)** NF-kB protein was expressed in the nucleus of hepatic nonparenchymal cells and injured hepatocytes in *iNOS−/−*/HFD mice at 48 weeks. **(E)** Confocal microscopic images showed that NF-kB-activated cells (red) were almost completely merged with F4/80-positive cells (green) in *iNOS−/−*/HFD mice. The nuclei were stained with 4′-diamidine-2′-phenylindole hydrocholoride (DAPI: blue). (Data are expressed as the mean ± SEM. * *p* < 0.05, represents significant difference between *iNOS+/+*/HFD mice and *iNOS−/−*/HFD mice).

Activated hepatic stellate cells (HSCs) were immunohistochemically stained with α-SMA antibodies of only *iNOS−/−*/HFD mice at 48 weeks (Figure 5A). Analysis of liver mRNA involved in activated HSCs promoting liver fibrosis revealed that liver interleukin1 (IL-1), tumor necrosis factor-alpha (TNF-α), transforming growth factor-beta (TGF-β), platelet-derived growth factor (PDGF), tissue inhibitor of metalloproteinase (TIMP)-1, TIMP-2 and collagen I were significantly up-regulated in *iNOS−/−*/HFD mice compared with *iNOS+/+*/HFD mice (Figure 5B).

As the up-regulated molecules are closely involved with inflammation and fibrosis, we therefore investigated their upstream transcriptional regulation. The activation of NF-kB in *iNOS−/−*/HFD in comparison to that in *iNOS+/+*/HFD mice was observed by EMSA analysis at 48 weeks (Figure 5C). The increased activation of NF-kB in hepatic nonparenchymal cells and injured hepatocytes in *iNOS−/−*/HFD mice, compared with that in *iNOS+/+*/HFD mice, was also confirmed using immunohistochemical staining (Figure 5D). To investigate the localization of NF-kB activation in the liver, we performed immunohistochemical staining with anti-phospho-NF-kB and anti-F4/80 antibodies as markers for macrophage and Kupffer cell lineages and anti-desmin antibodies as a marker for hepatic stellate cells in *iNOS+/+*/HFD and *iNOS−/−*/HFD mice. Confocal microscopic images showed that the NF-kB-activated cells were almost completely merged with the F4/80-positive cells only in the *iNOS−/−*/HFD mice (Figure 5E), although the NF-kB-activated cells were not merged with the desmin-positive cells in either group of mice (data not shown).

## Discussion

NAFLD is a common chronic liver disease displaying a wide spectrum of liver damage, ranging from simple steatosis to steatohepatitis, advanced fibrosis and cirrhosis. In the present study, we performed a follow-up study using HFD-induced NAFLD mouse models with or without the iNOS gene. Actually, we have previously reported that genetic variations in iNOS may influence the risk of NAFLD and liver fibrosis in NAFLD patients [24].

In our NAFLD model using HFD conditions, steatosis was observed after 10 weeks, and typical fibrosis was observed after 36–48 weeks of HFD feed [2,4].

In this study, systemic deficiency of iNOS showed the drastic promotion of hepatic fibrosis and inflammation (characteristics of NASH) after 48 weeks of HFD feed. However, the accumulation of lipids in iNOS-deficient mice livers after 48 weeks of HFD feed was less than that in wild-type mice livers. These results indicate that iNOS-derived NO in HFD conditions prevent hepatic fibrosis and inflammation, and the deficiency of iNOS strongly accelerates progression to NASH, independent of excessive lipid accumulation in the liver.

Generally, NO is produced by NOS, an enzyme that exists in 3 isoforms encoded by distinct genes. While neural NOS and endothelial NOS are constitutive isoforms, iNOS is not expressed under normal conditions but can be induced by cytokines and lipopolysaccharides in many cell types, such as hepatocytes, macrophages (including Kupffer cells), neutrophils, smooth muscle cells, and chondrocytes [25].

Increased NO generation during HFD conditions in our study is considered to be derived from iNOS, because iNOS knockout mice did not show any increase in NO metabolites during HFD conditions. Additionally, increased NO metabolites in liver is considered to be produced in liver, because the short half-time of NO in blood limits the transport of NO to distance tissues and the actions of NO are restricted to the site of production [26]. The up-regulation of iNOS was induced during HFD conditions, although the mechanisms involved in the up-regulation of iNOS during HFD conditions are unknown. Potential factors involved in iNOS induction in muscle and fat of HFD-fed obese mice may be the pro-inflammatory cytokines or NEFA [6]. Further investigations will be required to clarify the mechanisms.

Perreault M. et al. has reported that systemic deficiency of iNOS prevented the development of whole-body IR in mice fed a HFD [6]. Their observations seem at odds with the findings in our present study that iNOS-derived NO prevents NASH progression. However, in our model, the lipid accumulation in iNOS knockout mice livers was less than that of wild-type mice livers at 48 weeks during HFD conditions. Also, during the early stages during HFD conditions (10 weeks), whole-body IR was typically observed in wild-type mice rather than iNOS knockout mice—results similar to those of Perreault. Why was the discrepancy observed? Is iNOS-derived NO good or bad for the progression to NASH?

Based on the long-term observations (48 weeks), the deficiency of iNOS-derived NO during HFD conditions accelerates progression to NASH. In addition, fibrosis and inflammation were easily induced by a lack of NO, without excessive lipid accumulation in liver. This suggests that the pathogenesis of our long-term NASH model observations of iNOS-deficiency during HFD conditions may represent advanced NASH, while intrahepatic lipogenesis might also be suppressed. Rockey D.C. et al. suggested that the role of iNOS during hepatic injury and fibrosis varied with differences in the type of insult, duration and amount of NO actually generated in the liver [9]. In our present study, time-dependent and HFD-induced increases in NO generation in the liver were observed. Thus, the discrepancy observed in our study might be explained by the difference in duration and the amount of NO [9,27], in the same way that the conflicting effects of NO on the NAFLD/NASH liver [12,13] have been explained.

In an advanced-NASH mouse model at 48 weeks under HFD conditions, the high dose and long duration of the NO levels had a protective effect against hepatic inflammation and fibrosis. The reason why the iNOS-knockout mice fed an HFD had severer liver fibrosis despite the fact that they had less-severe hepatic steatosis may be an increase in serum NEFA and the liver NEFA content as a result of interactions among NO metabolites. Yamaguchi et al. and Soufi et al. reported that the accumulation of TG may be a protective mechanism preventing the progression of NAFLD/NASH and suggested that free fatty acid is a pathogenic mediator in the development of NASH, based on the progression of liver damage that occurs despite striking improvements in systemic insulin resistance and hepatic TG content [28,29].

The present study demonstrated that liver SREBP-1c, ChREBP, DGAT2, PPAR-α1 and MTP mRNA expression levels were all significantly decreased in iNOS-knockout mice fed an HFD for 48 weeks and that exhibited the progression of liver fibrosis, consistent with previous study results showing that the hepatic expression levels of genes involved in lipogenesis, fatty acid catabolism, and VLDL export in the liver were all suppressed during NASH pathogenesis [30]. In our study, a reduction in fat droplets in the liver during the unbalanced synthesis and discharge of lipids (“burned-out NASH”) might have occurred in the iNOS-deficient mouse at 48 weeks under HFD conditions.

In both the early-NASH mouse model at 10 weeks and the advanced-NASH mouse model at 48 weeks under HFD conditions, the long duration of the NO levels associated with either dose had an accelerative effect on systemic insulin resistance, although the NO conditions did not affect the local intrahepatic insulin resistance. Yamaguchi et al. reported a discrepancy between the progression of liver fibrosis and the striking improvement in systemic insulin resistance in mice fed a methionine and choline diet that were treated with DGAT2 antisense oligonucleotide; however, a detailed mechanism is not obvious [28].

The ob/ob mice lacking iNOS showed an improvement in energy balance because of a decrease in food efficiency arising from an increase in thermogenesis, although we could not perform metabolic cage experiments [31].

To clarify the mechanisms of hepatic fibrosis and inflammation induced by iNOS-derived NO deficiency during HFD conditions, we hypothesized that activation of NF-kB, which controls transcription of various pro-inflammatory genes, including cytokines, might be promoted by the iNOS deficiency. Our data showed an increase in expression levels of various cytokines that are transcriptionally controlled by NF-kB in the iNOS-deficient mouse liver. Liver NF-kB activity has previously been shown to be correlated with the pathogenesis of NASH [32], and biphasically regulated by NO [27]. The NO mechanism involved to bring about biphasic regulation of NF-kB activity is currently under investigation. NO induces and stabilizes the inhibitor of kappaBα (IkBα), which prevents nuclear location of NF-kB [33]. Connelly et al. reported that the NO donor produced a concentration-dependent influence on NF-kB activity and that NF-kB was enhanced at certain concentrations of NO followed by inhibition at a higher concentration [27]. Our EMSA results showed that the activation of liver NF-kB was significantly enhanced by the deficiency of iNOS-derived NO in the liver during HFD conditions, and confocal microscopic images showed that NF-kB-activated cells were almost consistent with the location of Kupffer cells. Therefore, in our NASH model, increased liver NO concentrations of wild-type mice might inhibit NF-kB activity in Kupffer cells. In contrast, iNOS deficiency under the same conditions might activate NF-kB in Kupffer cells, compared with the situation in wild-type mice. Further investigation is required to determine whether the activation of NF-kB in Kupffer cells might be one of the mechanisms involved in the progression of hepatic inflammation.

The main limitations of this study are related to its long-term study design and its inability to exclude the effect of aging on the results. Aging-associated changes in liver diseases have been previously reported with or without iNOS [34-36]. Aging may be a risk factor for metabolic syndrome and diet-induced steatohepatitis and might be accompanied by an increase in iNOS expression, consistent with our results shown in Figure 3F.

## Conclusions

**Figure 6:**
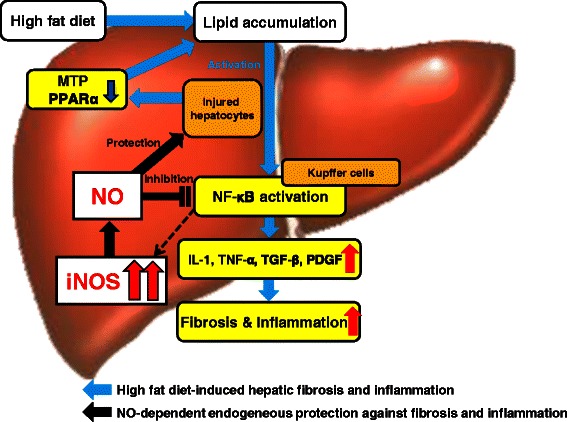
**Scheme.** iNOS-derived NO may play a protective role against the progression to NASH under HFD conditions by preventing fibrosis and inflammation mediated by NF-kB activation in Kupffer cells.

In conclusion, iNOS-derived NO may play a protective role against the progression to NASH under HFD conditions by preventing fibrosis and inflammation mediated by NF-kB activation in Kupffer cells. A lack of iNOS-derived NO accelerates progression to NASH, without excessive lipid accumulation, despite promoting systemic IR in the chronic NASH experimental model induced by long-term HFD feeding (Figure 6).

## Abbreviations

NASH: Non-alcoholic steatohepatitis
iNOS: inducible nitric oxide synthase
NO: Nitric oxide
HFD: High fat diet
AMPK: AMP-activated protein kinase
PPAR-α: Peroxisome proliferators-activated receptor alpha
NAFLD: Non-alcoholic fatty liver disease
IR: Insulin resistance
NAFL: Non-alcoholic fatty liver
VLDL: Very low-density lipoprotein
MTP: Microsomal triglyceride transfer protein
BD: Basal diet
ALT: Alanine aminotransferase
TG: Triacylglycerol
NEFA: Nonesterified fatty acid
HOMA-IR: Homeostasis model assessment of IR
ITT: Insulin torelance test
H&E: Hematoxylin and eosin
α-SMA: α-Smooth Muscle antibody
NF-kB: Nuclear factor kappa B
NAS: NAFLD activity score
EMSA: Electrophoretic mobility shift assays
SREBP-1c: Sterol regulatory element binding protein 1c
ChREBP: Carbohydrate response element binding protein
DGAT2: Diacylglycerol acyltransferase2
HSCs: Hepatic stellate cells
IL-1: Interleukin1
TNF-α: Tumor necrosis factor-alpha
TGF-β: Transforming growth factor-beta
PDGF: Platelet-derived growth factor
TIMP: Tissue inhibitor of metalloproteinase
IkBα: Inhibitor of kappaBα
